# Effects of flexible-dose fesoterodine on overactive bladder symptoms and treatment satisfaction: an open-label study

**DOI:** 10.1111/j.1742-1241.2009.02035.x

**Published:** 2009-04

**Authors:** J-J Wyndaele, E R Goldfischer, J D Morrow, J Gong, L-J Tseng, Z Guan, M-S Choo

**Affiliations:** 1 Department of Urology, Universiteit en Universitair Ziekenhuis AntwerpenAntwerp, Belgium; 2 Hudson Valley Urology, PCPoughkeepsie, NY, USA; 3 Pfizer IncNew York, NY, USA; 4 Department of Urology, Asan Medical Center, University of Ulsan College of MedicineSeoul, Republic of Korea

## Abstract

**Aims:**

To evaluate the efficacy and tolerability of flexible-dose fesoterodine in subjects with overactive bladder (OAB) who were dissatisfied with previous tolterodine treatment.

**Methods:**

This was a 12-week, open-label, flexible-dose study of adults with OAB (≥ 8 micturitions and ≥ 3 urgency episodes per 24 h) who had been treated with tolterodine (immediate- or extended-release) for OAB within 2 years of screening and reported dissatisfaction with tolterodine treatment. Subjects received fesoterodine 4 mg once daily for 4 weeks; thereafter, daily dosage was maintained at 4 mg or increased to 8 mg based on the subject’s and physician’s subjective assessment of efficacy and tolerability. Subjects completed 5-day diaries, the Patient Perception of Bladder Condition (PPBC) and the Overactive Bladder Questionnaire (OAB-q) at baseline and week 12 and rated treatment satisfaction at week 12 using the Treatment Satisfaction Question (TSQ). Safety and tolerability were assessed.

**Results:**

Among 516 subjects treated, approximately 50% opted for dose escalation to 8 mg at week 4. Significant improvements from baseline to week 12 were observed in micturitions, urgency urinary incontinence episodes, micturition-related urgency episodes and severe micturition-related urgency episodes per 24 h (all p< 0.0001). Approximately 80% of subjects who responded to the TSQ at week 12 reported satisfaction with treatment; 38% reported being very satisfied. Using the PPBC, 83% of subjects reported improvement at week 12 with 59% reporting improvement ≥ 2 points. Significant improvements from baseline (p< 0.0001) exceeding the minimally important difference (10 points) were observed in OAB-q Symptom Bother and Health-Related Quality of Life (HRQL) scales and all four HRQL domains. Dry mouth (23%) and constipation (5%) were the most common adverse events; no safety issues were identified.

**Conclusion:**

Flexible-dose fesoterodine significantly improved OAB symptoms, HRQL, and rates of treatment satisfaction and was well tolerated in subjects with OAB who were dissatisfied with prior tolterodine therapy.

**What’s known**

Fixed-dose clinical trials have shown that fesoterodine 4 or 8 mg once daily significantly improves bladder diary variables and measures of health-related quality of life compared with placebo and that fesoterodine is generally well tolerated.

**What’s new**

This is the first flexible-dose trial of fesoterodine. Dose escalation was based on subject and physician assessment of efficacy and tolerability, which mimics clinical practice. Flexible-dose fesoterodine was associated with a high rate of self-reported treatment satisfaction, produced significant improvements in bladder diary variables and measures of symptom bother and health-related quality of life, and was generally well tolerated.

## Introduction

Overactive bladder (OAB) is defined by urgency, with or without urgency incontinence, usually with increased daytime frequency and nocturia (1). OAB is a chronic condition affecting 12–17% of the adult population in Europe and North America (2–4). Although antimuscarinic agents are the pharmacological mainstay of OAB treatment (5,6), efficacy and tolerability vary among agents and patients (7).

Fesoterodine is an antimuscarinic developed for the treatment of the symptoms of OAB. Fesoterodine is rapidly and extensively converted by non-specific esterases to its active metabolite, 5-hydroxymethyl tolterodine (5-HMT), which is also the active metabolite of tolterodine (8). The oxidation of tolterodine to 5-HMT is mediated by cytochrome P450 (CYP) 2D6 in the liver, and because there is substantial interindividual variability in CYP2D6 metabolic activity, extensive and poor metabolisers have markedly different proportions of plasma tolterodine to 5-HMT following tolterodine administration (9). The esterases that convert fesoterodine to 5-HMT do not exhibit genotypic variations and they are not known to be involved in any drug–drug interactions. Thus, the pharmacokinetic variability among individuals treated with fesoterodine is lower. Moreover, all antimuscarinic activity following fesoterodine administration is due to 5-HMT, whereas both tolterodine and 5-HMT contribute to the antimuscarinic activity of tolterodine.

Two previous randomised, double-blind, placebo-controlled, fixed-dose phase 3 studies demonstrated that fesoterodine 4 or 8 mg once daily (qd) significantly improves OAB symptoms and measures of health-related quality of life (HRQL) compared with placebo (10–12). Moreover, compared with subjects receiving placebo, a significantly greater proportion of subjects receiving fesoterodine 4 or 8 mg reported a Treatment Response, a yes/no variable derived from the validated four-point Treatment Benefit Scale (13). The availability of the two doses of fesoterodine provides an opportunity to establish an optimal balance between efficacy and tolerability in individual patients. However, the fixed-dose fesoterodine data currently available in the literature do not provide clinicians with information regarding efficacy and tolerability with flexible dosing to guide their use in clinical practice. Furthermore, data on the efficacy and tolerability of fesoterodine in patients who are not satisfied with tolterodine for OAB would also be relevant to clinical practice.

The primary objective of this open-label study was to assess the effect of a flexible-dosing regimen of fesoterodine on OAB symptoms and treatment satisfaction in subjects with OAB who were dissatisfied with previous tolterodine or tolterodine extended release (ER) treatment. Secondary objectives included evaluating the effect on measures of HRQL and other patient-reported outcomes as well as the safety and tolerability of fesoterodine therapy.

## Subjects and methods

### Study design

In this 12-week, multicentre, open-label, single-arm, flexible-dose study, the effect of fesoterodine on OAB symptoms and treatment satisfaction was assessed in adult subjects with OAB who were dissatisfied with previous tolterodine or tolterodine ER therapy. This study was conducted at 80 centres worldwide, with centres in Asia, Europe, and North and Central America. The study was conducted in accordance with the Good Clinical Practice guidelines and the Declaration of Helsinki. The protocol was approved by the respective Institutional Review Boards/Independent Ethics Committees, and all subjects provided written informed consent before the start of the study.

Baseline OAB symptoms were assessed using a 5-day bladder diary during a 2-week screening period, before which any previous antimuscarinic medication was stopped. Subjects rated the sensation associated with each micturition or urgency urinary incontinence (UUI) episode in the diary using the five-point Urinary Sensation Scale (USS; 1 = no urgency, 2 = mild urgency, 3 = moderate urgency, 4 = severe urgency, 5 = UUI) (14). All enrolled subjects were treated for 4 weeks with fesoterodine 4 mg qd, taken in the morning. At week 4, dosage could either be maintained at fesoterodine 4 mg qd or increased to 8 mg qd for the remaining 8 weeks of the study. Consistent with clinical practice, the decision to maintain or increase the dose of fesoterodine was based on a discussion between the subject and the investigator regarding their subjective assessment of treatment efficacy and tolerability.

## Subjects

Eligible subjects were men and women aged ≥ 18 years with self-reported OAB symptoms for ≥ 3 months before screening with a mean micturition frequency of ≥ 8 micturitions per 24 h and mean number of urgency episodes ≥ 3 per 24 h in a 5-day bladder diary at baseline (urgency episodes were defined as those with a USS rating ≥ 3). Subjects had to rate their bladder condition as causing at least ‘some moderate problems’ on the Patient Perception of Bladder Condition (PPBC) questionnaire at baseline. Subjects were also required to have been treated with tolterodine or tolterodine ER for OAB within 2 years of screening and to report being ‘somewhat dissatisfied’ or ‘very dissatisfied’ with tolterodine treatment on the Treatment Satisfaction Question (TSQ), a single item from the validated Overactive Bladder Satisfaction Questionnaire (15); subjects are asked how satisfied they are with their OAB medication and respond on a five-point Likert scale. Subjects who had received prior OAB treatment with ≥ 3 antimuscarinics (including tolterodine) within 12 months, or who had neurogenic bladder, a history of acute urinary retention requiring catheterisation, predominant stress urinary incontinence, significant pelvic organ prolapse, lower urinary tract surgery within 6 months, significant hepatic or renal function impairment, or any contraindication to fesoterodine usage, were excluded from the study. Subjects’ reasons for dissatisfaction with previous tolterodine treatment were not collected.

### Assessments

To assess efficacy, subjects completed 5-day bladder diaries at baseline and at 12 weeks. Subjects recorded the time of every micturition and rated the sensation associated with each micturition using the five-point USS. Subjects rated satisfaction with fesoterodine treatment at week 12 using the TSQ. Subjects also completed several validated OAB-specific questionnaires at baseline and week 12, including the PPBC (16), the Urgency Perception Scale (UPS) (17) and the Overactive Bladder Questionnaire (OAB-q) (18). The PPBC is a single-item six-point instrument used by subjects to rate the severity of their bladder-related problems**:**‘My bladder causes me no (1), very minor (2), minor (3), moderate (4), severe (5) or many severe (6) problems’. The UPS is a three-point scale; response options include: ‘usually not able to hold urine’ (1), ‘usually able to hold urine if I go to the toilet immediately’ (2) and ‘usually able to finish what I am doing before going to the toilet’ (3). The OAB-q comprises an eight-item Symptom Bother scale and a 25-item HRQL scale with 4 domains (Concern, Coping, Sleep and Social Interaction). Safety and tolerability were assessed.

Primary end-points were change from baseline to week 12 in number of micturitions, number of UUI episodes (among subjects with a baseline UUI > 0), and number of micturition-related urgency episodes (defined as those with a USS rating ≥ 3) per 24 h and the percentage of subjects reporting treatment satisfaction at week 12 (‘very satisfied’ or ‘somewhat satisfied’ on the TSQ).

Secondary bladder diary end-points included change from baseline to week 12 in nocturnal micturitions, severe micturition-related urgency episodes (defined as those with a UUS rating of ≥ 4), and frequency-urgency sum (defined as the sum of all USS ratings) per 24 h. Additional secondary end-points included change from baseline in PPBC, UPS and OAB-q scores at week 12.

### Safety and tolerability

All adverse events (AEs), whether directly observed by the investigator, reported by the subject or resulting from investigator questioning of the subject regarding their tolerance of study treatment, were recorded during the entire study period. The causality (based on the investigator’s assessment), severity and outcome of each AE were recorded.

### Statistical analyses

A sample size of 400 subjects was calculated to provide a 5% level of precision in the 95% CI for the percentage of subjects reporting treatment satisfaction at week 12. The other primary end-points were powered for 95%, resulting in an overall power of 85%. Statistical analyses of all efficacy variables at week 12 were performed using the full analysis set (i.e. all subjects who took ≥ 1 dose of study drug and contributed data to at least one baseline or postbaseline efficacy assessment). The last valid postbaseline observation was carried forward to handle missing efficacy data at week 12. Descriptive statistics and two-sided paired *t*-tests at the 5% significance level were used to analyse efficacy end-points. Tolerability analyses were performed based on the data from all subjects who took at least one dose of study drug. Subjects were not stratified by titration status for the assessment of efficacy or tolerability.

## Results

### Subjects

**Figure 1:**
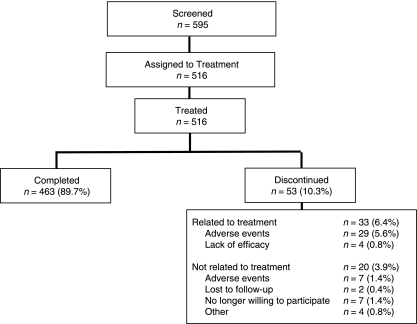
Subject disposition

Subject disposition is shown in Figure 1. Subject demographics are shown in Table 1. Most subjects were women (77%) and white (77%) and the mean age was 60 years; three-quarters of the women were postmenopausal. A total of 256 (50%) subjects reported at least one episode of UUI at baseline, including nearly one-fifth (*n*= 22; 19%) of the men and three-fifths (*n*= 234; 59%) of the women. In addition to prior treatment with tolterodine or tolterodine ER, 216 subjects (42%) had received ≥ 1 other antimuscarinic prior to study enrolment. Notably, 67% of all male subjects in the study reported a history of benign prostatic hyperplasia. Eleven subjects included in this study did not report being dissatisfied with prior tolterodine treatment at the beginning of the study; inclusion of these 11 major protocol violators did not materially affect the study results.

**Table 1:** Baseline demographics and clinical characteristics*

	Men	Women	Total
Gender, *n* (%)	118 (23)	398 (77)	516
Age, mean ± SD (years)	64 ± 12	58 ± 14	60 ± 14
Range	19–83	19–90	19–90
Race, *n* (%)			
White	92 (78)	303 (76)	395 (77)
Black	5 (4)	5 (1)	10 (2)
Asian	21 (18)	76 (19)	97 (19)
Other	0	14 (4)	14 (3)
Postmenopausal,*n* (%)	NA	304 (76)	NA
Past or presentBPH, *n* (%)	79 (67)	NA	NA
Incontinent atbaseline, *n* (%)†	22 (19)	234 (59)	256 (50)

* Safety analysis set (equivalent to full analysis set).

† UUI > 0 episodes on baseline diary.

BPH, benign prostatic hyperplasia; NA, not applicable.

Of the 516 subjects who received at least 1 dose of study drug and had at least one postbaseline assessment, 255 (50%, excluding two subjects who started with 8 mg) opted for dose escalation to fesoterodine 8 mg at week 4. The remaining subjects continued receiving the 4-mg dose.

### Efficacy

#### OAB symptoms

**Figure 2:**
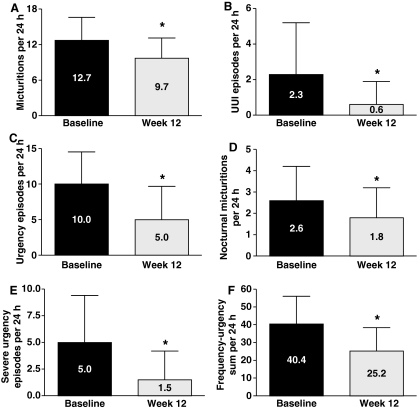
Number of (A) micturitions, (B) UUI episodes (for subjects reporting > 0 UUI episodes at baseline), (C) urgency episodes, (D) nocturnal micturitions, (E) severe urgency episodes (for subjects reporting > 0 severe urgency episodes at baseline) and (F) frequency-urgency sum per 24 h at baseline and at 12 weeks of fesoterodine treatment. Data shown are mean ± standard deviation. UUI = urgency urinary incontinence. *p < 0.0001 vs. baseline

Statistically significant improvements from baseline to week 12 were observed in mean number of micturitions, UUI episodes and urgency episodes (p< 0.0001 for all comparisons; Figure 2). Statistically significant improvements in nocturnal micturitions, severe urgency episodes and frequency-urgency sum were also observed at week 12 (p< 0.0001 for all comparisons; Figure 2). The corresponding median % change from baseline to week 12 was −22% for micturition frequency, −100% for UUI episodes, −57% for urgency episodes, −31% for nocturnal micturitions and −94% for severe urgency episodes.

#### Patient-reported outcomes

**Figure 3:**
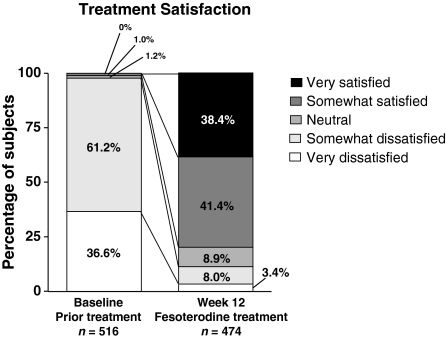
Percentage of subjects reporting Treatment Satisfaction with prior treatment (baseline) or current treatment (week 12)

**Figure 4:**
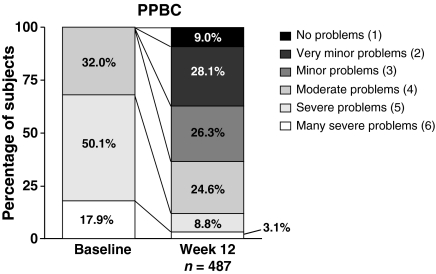
Response distribution on the PPBC questionnaire at baseline and after 12 weeks of fesoterodine treatment. PPBC = Patient Perception of Bladder Condition

At 12 weeks, 80% of subjects who responded to the TSQ reported being satisfied with fesoterodine treatment, with 38% of subjects being ‘very satisfied’ (Figure 3). Mean PPBC scores improved significantly from 4.9 at baseline to 3.1 at week 12 (p< 0.0001). By week 12, 83% of subjects reported improvement on the PPBC, with 59% of subjects reporting improvement ≥ 2 points. The proportion of subjects reporting severe or many severe problems was reduced from 68% at baseline to 12% after 12 weeks, whereas the proportion reporting no problems, very minor problems or minor problems was increased from zero at baseline (as required by the inclusion criteria) to 63% at 12 weeks (Figure 4).

**Figure 5:**
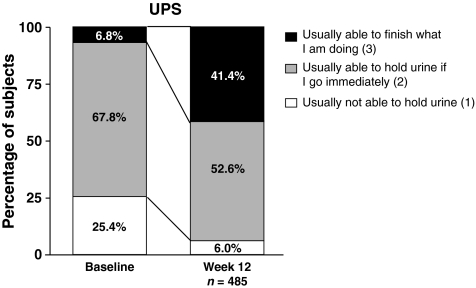
Response distribution on the UPS at baseline and after 12 weeks of fesoterodine treatment. UPS = Urgency Perception Scale

Mean UPS scores improved significantly from 1.8 at baseline to 2.4 at week 12 (p< 0.0001). UPS scores improved in 49% of subjects, deteriorated (*post hoc* analysis) in 2%, and were unchanged in the remaining subjects. The proportion of subjects who reported that they were usually not able to hold their urine was reduced from 25% at baseline to 6% after 12 weeks. The proportion of subjects who reported being able to finish what they were doing before going to the toilet was increased from 6.8% at baseline to 41% after 12 weeks (Figure 5).

**Figure 6:**
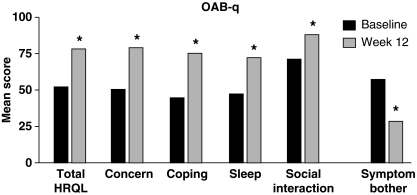
Overactive Bladder Questionnaire (OAB-q) scores for total HRQL, the four HRQL domains, and Symptom Bother are shown at baseline and after 12 weeks of fesoterodine treatment. A positive score change in total HRQL and its domains indicates improvement; a negative score change in Symptom Bother indicates improvement. HRQL = health-related quality of life. *p< 0.0001 vs. baseline

The mean change in OAB-q Symptom Bother score (29-point improvement) from baseline to week 12 was statistically significant (p< 0.0001; Figure 6). Mean changes in total HRQL (26-point improvement) and all four HRQL domain (Concern, 29-point improvement; Coping, 31-point improvement; Sleep, 25-point improvement; Social Interaction, 17-point improvement) scores were also statistically significant at 12 weeks, compared with baseline (p< 0.0001; Figure 6). The improvements for all scales and domains were well above the minimally important difference of 10 points, indicating that these changes were clinically meaningful (19).

#### Safety and tolerability

Dry mouth (23%) and constipation (5%) were the most frequently reported AEs (Table 2); most of these were mild or moderate in severity. Urinary retention requiring catheterisation was reported by one woman receiving fesoterodine 8 mg who withdrew from the study. Two women receiving the 4-mg dose were reported to have urinary retention not requiring catheterisation; one withdrew from the study. No cases of urinary retention occurred in men. The overall withdrawal rate was 10% and the rate of withdrawal due to treatment-emergent AEs regardless of causality was 7%. There were no deaths during the study; nine subjects (< 2%) reported serious AEs, none of which was deemed to be treatment related.

**Table 2:** Treatment-emergent adverse events reported by ≥ 1% of subjects (all causality)

		Severity, *n*		
Adverse event, *n* (%)	*n*= 516	Mild	Moderate	Severe
Dry mouth	120 (23.3)	98	16	6
Constipation	25 (4.8)	16	7	2
Headache	19 (3.7)	14	4	1
Diarrhoea	12 (2.3)	10	2	0
Abdominal pain, upper	11 (2.1)	6	3	2
Dizziness	6 (1.2)	5	1	0
Dry eye	6 (1.2)	6	0	0
Dysuria	6 (1.2)	3	1	2
Nausea	6 (1.2)	5	1	0

## Discussion

This is the first phase 3b study of fesoterodine and the first fesoterodine study to employ flexible dosing. The results presented here show that flexible-dose fesoterodine significantly improved several bladder diary variables as well as subjects’ assessment of their bladder-related problems, urgency, symptom bother and HRQL in subjects who reported dissatisfaction with previous tolterodine treatment. Additionally, approximately 80% of respondents in the current study reported satisfaction with fesoterodine treatment at week 12. These findings were expected considering the results of two previous fixed-dose placebo-controlled phase 3 clinical trials that demonstrated significantly greater improvements in bladder diary variables and HRQL measures and significantly greater rates of self-reported treatment response in subjects who received fesoterodine 4 or 8 mg compared with those who received placebo (10–12). Fesoterodine was well tolerated.

Approximately half of the subjects in this study opted to escalate their fesoterodine dose to 8 mg at week 4. This decision was based on discussion with the investigator about efficacy and tolerability, as assessed subjectively by the subject and investigator. The availability of two doses allows for individualisation of patient care. A pooled, *post hoc* analysis of the two phase 3 trials showed that fesoterodine 8 mg was significantly more efficacious than fesoterodine 4 mg in improving UUI episodes, mean voided volume per micturition, continent days per week (extrapolated from 3-day diaries), and subject-reported Treatment Response at week 12, indicating an apparent efficacy dose–response effect on these end-points (20). Thus, dose escalation may allow for improved outcomes in those patients who report good tolerability and desire greater symptom relief. However, we did not assess whether there was a dose–response effect in this analysis.

This was an open-label study. Open-label trials reflect clinical practice; however, they have inherent limitations. For example, open-label studies are unable to account for placebo effects, which are often substantial in randomised placebo-controlled clinical trials of antimuscarinics for OAB (21). Another limitation is that this study was not designed *a priori* to compare in its primary analysis efficacy and tolerability in subjects who received the 4-mg dose throughout the study with subjects who escalated to the 8-mg dose at week 4, and comparisons by dose are limited by the flexible dosing design. Differences in efficacy and tolerability between subjects who did and did not opt for dose escalation will be assessed at time points before and after dose escalation in *post hoc* analyses. Additionally, we did not capture reasons why subjects did or did not opt for dose escalation. However, this study is meant to reflect real-world clinical conditions. Presumably, this study provides information regarding fesoterodine efficacy and tolerability that is more relevant to real-world clinical practice than fixed-dose studies, because the final dose each subject received was determined by a discussion between the subject and investigator about efficacy and tolerability rather than random assignment. As in real-world clinical practice, the reasons underlying the decision of whether or not to increase the dose likely varied between individuals but reflected optimisation of the balance between efficacy and tolerability. Further, detailed information about the reason for dissatisfaction with previous tolterodine treatment was not collected, which limited our ability to determine what aspects of fesoterodine contributed to the high rate of treatment satisfaction (i.e. efficacy and/or tolerability) in this population or predict which reasons might lead to dissatisfaction.

## Conclusions

When used with a flexible-dosing regimen in this open-label study, fesoterodine significantly improved OAB symptoms and HRQL measures and reduced OAB-related symptom bother in subjects with OAB who had been dissatisfied with previous tolterodine or tolterodine ER treatment. Approximately 80% of subjects reported treatment satisfaction and about 50% of subjects opted to receive the higher 8-mg dose after initial 4-mg treatment. Fesoterodine was well tolerated.
